# Authors' Response to Hogan

**DOI:** 10.1371/journal.pmed.0030415

**Published:** 2006-09-26

**Authors:** Bart L Haagmans, Albert D. M. E Osterhaus

In response to our Perspective on nonhuman primate models for SARS [1], which accompanied the article by Lawler et al. [2], Robert Hogan questions the usefulness of nonhuman primates as good models for SARS [3].

As demonstrated by several groups, SARS coronavirus (SARS-CoV) replicates to high titers in the respiratory tract of a surprisingly broad range of animal species, albeit showing remarkable differences in cell tropism. We have argued that efficient infection of type 1 and 2 pneumocytes as seen in macaques and humans—most likely due to the similarities in the spike protein binding domains of the host SARS-CoV receptor ACE2—is a prerequisite for the SARS-CoV infection–induced pathology observed in humans. So far there is no strong evidence that a similar tropism is observed in other animal species.

However, the tropism of SARS-CoV for pneumocytes may not suffice to induce severe clinical signs in primates. In case of a resolving infection in macaques, pathological changes are evident four to six days after infection and may have become inapparent by days 12–14. In addition, in young adult humans, SARS-CoV infection generally causes relatively mild disease. Viral sequence differences in the SARS-CoV isolate HKU 39849 obtained from and distributed to several groups by Peiris et al. [4], as compared to other strains used, may determine the outcome of SARS-CoV infection in macaques. Furthermore, recent experiments from our group indicate that clinical signs after infection with this virus are more likely to occur in aged macaques (unpublished data). Clinical signs observed in our earlier experiments were largely characterised by lethargy, whereas respiratory distress was observed in one animal [5,6].

The apparent differences observed in clinical outcome of the infection in macaques, which is similar to the outcome in humans, may limit the utility of SARS-CoV–infected macaques as a model for severe SARS. However, none of the other models available produces clinical disease related to respiratory distress, except for ferrets that are inoculated with the HKU 39849 virus intratracheally [7]. Therefore, we feel that the macaque model may indeed provide important clues to the pathogenesis of SARS. As also argued by Subbarao and Roberts [8], there is no single preferred model for SARS and combining the data obtained in different animal models may help to solve the question of why SARS is so devastating in some humans. This also holds true for the development of effective vaccines and other intervention strategies against possible future SARS-CoV outbreaks which may be caused by genetically quite distinct viruses.
